# A low-cost continuous rumen simulation system for sustainable in vitro fermentation and feed evaluation in ruminant production

**DOI:** 10.1016/j.mex.2025.103581

**Published:** 2025-08-22

**Authors:** Harneet Kour, Raman Malik, Chander Datt, Parul Rana

**Affiliations:** Animal Nutrition Division, ICAR-National Dairy Research Institute, Karnal-132001, India

**Keywords:** Continuous bioreactor, Rumen simulation, Microbial fermentation, In vitro digestion, Animal nutrition, Methane mitigation, Low-cost system

## Abstract

In vitro simulation of rumen fermentation is critical for improving feed efficiency, assessing dietary interventions, and supporting methane mitigation strategies in ruminant production systems. However, existing fermentation platforms are often expensive, technically complex, or poorly suited for long-term microbial viability under near-rumen conditions—especially in resource-limited settings. This study presents the development and validation of a modular, low-cost **Continuous Rumen Bioreactor (CRB)** engineered to replicate key physiological parameters of the rumen, including temperature control (39–40 °C), continuous buffering via artificial saliva infusion, anaerobic regulation, and simulated motility through mixing pumps. A novel feed cartridge system was incorporated to facilitate uninterrupted substrate exchange during multi-week incubations. The system was tested using rumen liquor from buffalo calves and evaluated for fermentation consistency, pH stability, gas production, and dry matter degradation. Results demonstrated that the CRB maintained functional microbial activity over extended durations, providing reliable and reproducible fermentation outcomes. The system offers a scalable and accessible alternative to conventional in vitro models and supports practical applications in feed evaluation, microbial enrichment, and enteric methane mitigation strategies. Its affordability and adaptability make it particularly relevant for use in developing countries and decentralized agricultural research systems.

• A low-cost, modular Continuous Rumen Bioreactor (CRB) was developed to simulate rumen fermentation over extended durations.
• Maintains key rumen conditions (temperature, pH, anaerobiosis, and motility) and supports consistent microbial activity.
• Suitable for feed evaluation, microbial enrichment, and methane mitigation research, especially in resource-limited settings.

## Graphical abstract

**Figure:**
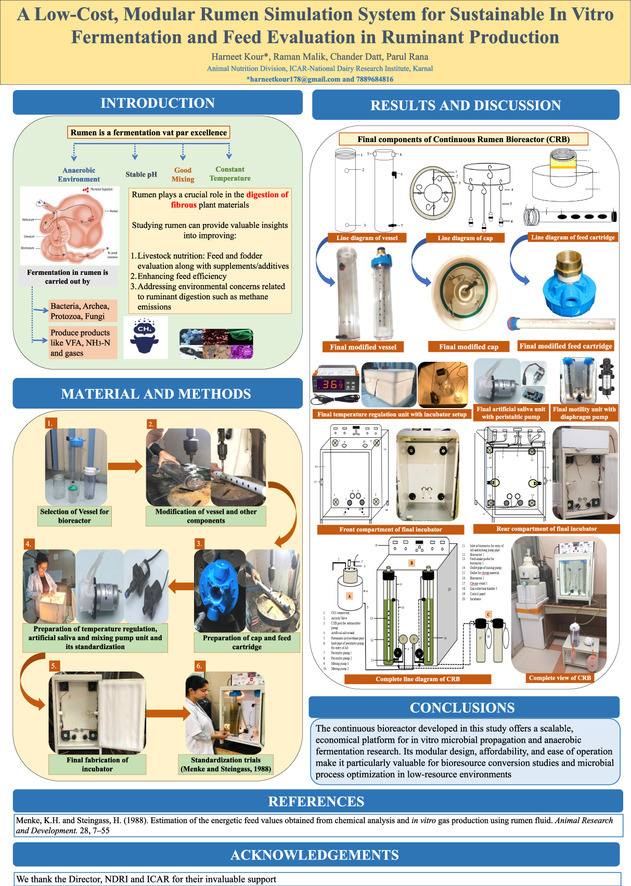

**Specifications Table:**

**Table:** 

**Subject area**	**Animal Science / Microbiology**
More specific subject area	Ruminant Nutrition, In Vitro Fermentation
Method name	Continuous Rumen Bioreactor System
Name and reference of original method	Menke and Steingass (1988), In Vitro Gas Production Technique
Resource availability	Available upon request from corresponding author

## Background

Efficient fermentation within the rumen plays a critical role in ruminant nutrition, driven by a highly diverse microbial population capable of degrading fibrous plant material into volatile fatty acids, microbial protein, and gases such as methane. Methane emissions from ruminants not only represent a loss of feed energy but also contribute significantly to greenhouse gas emissions, with methane having a global warming potential many times higher than carbon dioxide. In light of the increasing emphasis on sustainable livestock production, there is a growing need to investigate and optimize feed strategies that enhance microbial efficiency and mitigate enteric methane emissions.

To study these microbial processes, in vitro fermentation systems have long been used as alternatives to in vivo trials, offering controlled environments that reduce ethical concerns, cost, and variability. Traditional batch systems and more advanced continuous fermentation models like RUSITEC have contributed substantially to our understanding of rumen metabolism. However, these systems often fall short in simulating the dynamic and complex conditions of the rumen over prolonged periods. Common limitations include mechanical complexity, inadequate temperature regulation, lack of real-time sampling options, high setup costs, and declining microbial viability during extended incubation. Furthermore, many of these systems are not easily scalable or adaptable for routine use, particularly in low-resource settings.

There is a clear need for a more accessible, cost-effective, and modular system that can sustain microbial populations and fermentation activity under rumen-like conditions over extended durations. Such a system would not only support ongoing research in methane mitigation but also enable microbial enrichment and evaluation of feed interventions in a reproducible and scalable manner. In vivo studies have demonstrated that inclusion of alternative carbohydrate sources such as cassava can influence growth performance and rumen fermentation profiles in buffaloes [1]. Amirul et al. [1], through a meta-analysis, reported consistent improvements in performance and alterations in rumen fermentation patterns, highlighting the need for controlled laboratory systems such as the Continuous Rumen Bioreactor (CRB) to explore such dietary interventions before field application.

This study hypothesizes that a simplified, thermostatically controlled Continuous Rumen Bioreactor (CRB) can effectively replicate key physiological conditions of the rumen—anaerobiosis, mixing, and thermal stability—while allowing long-term incubation and real-time sampling. The primary objectives were (1) to design and validate a low-cost bioreactor capable of supporting extended in vitro fermentation, (2) to evaluate microbial viability and fermentation efficiency using various inocula and substrates, and (3) to establish its potential as a practical platform for methane mitigation studies and microbial propagation. This work contributes to the state-of-the-art by offering an engineered yet accessible system that bridges the gap between laboratory feasibility and field-relevant rumen simulation technologies.

## Material details

The present study was conducted at Animal Nutrition division, National Dairy Research Institute, Karnal. A brief description of the experimental techniques and procedures of analysis adopted during the present study are reported in this chapter.

### Bioreactor vessel

Various types of vessels of different sizes and dimensions were considered for the preparation of the bioreactor. The objective was to utilize readily available resources rather than fabricating a custom vessel from scratch, thereby reducing both costs and time. This approach also aims to facilitate maintenance in the later stages, ensuring that resource availability will not pose a challenge. Two sizes of vessels (1 L and 2.5 L) made from acrylic-styrene were selected and modified for fermentation. Modifications included:

• Drilled and threaded ports for continuous buffer inflow and chyme outflow
• Gas-tight fittings using O-rings and Teflon tape
• Cap with modular feed addition unit (removable cartridge with nylon bags)

### Temperature regulation system

To maintain optimal environmental conditions for rumen microbial growth, a precise temperature regulation system was installed in bioreactor. The temperature control unit involved integrating components such as thermostat sensors, heating and air circulation unit. These components were selected based on their reliability, accuracy, and compatibility with the bioreactor system. The unit was designed to simulate rumen temperature at 39±1 °C by continuously monitoring the bioreactor's internal temperature. Necessary adjustments were made to ensure consistent thermal conditions.

### Artificial saliva delivery and flow control

An artificial delivery system was designed to ensure precise and controlled input of artificial saliva into the bioreactor vessel. The system comprised of peristaltic pump, 3-way valves and tubing. It was calibrated for accurate and consistent delivery of artificial saliva based on the requirements of the rumen fermentation. Components were selected based on their durability, chemical resistance, and compatibility with the bioreactor vessel. The system was programmed to operate in sync with the bioreactor's real-time monitoring controls, allowing adjustments of delivery rates to maintain optimal culture conditions throughout the experiment as per requirement.

A distilled water container (15 L volume) made up of polycarbonate was selected as artificial saliva container. It was modified to maintain a sterile and anaerobic environment. Holes were drilled into the cap of the container, with each hole serving a specific function: one for the entry of the CO₂ pipe, which allowed for intermittent CO₂ flushing; one for an air safety valve to prevent pressure buildup inside the tank; and one for a USB port used to power the submersible pump. The submersible pump was used for continuous mixing of artificial saliva contents to prevent the settling of salts at the bottom. To fulfill these requirements the artificial saliva was prepared following the composition described by McDougall [2].

### Motility simulation system

For simulating the rumen conditions, one of the important steps was to include motility which mimics the rumen motility pattern. This feature is lacking in previous techniques such as in vitro gas production technique [3] making it a unique and important feature of our developed apparatus. For mimicking the rumen motility, various options were explored like vibrator, submersible pumps, centrifugal pumps and diaphragm pump.

### Gas collection and measurement

A gas leakage test was conducted in bioreactor vessel. This was done to ensure the gas-tight integrity of the bioreactor, connectors, and the gas collection assemblies prior to the fermentation trials. The test involved two steps: a water immersion test and a pressure test using an air pump.

### Preliminary fermentation trials

To determine the maximum operating period of bioreactor, two trials were conducted on 1 L and 2.5 L vessel respectively. Rumen liquor was used as medium in both trials and substrate consisted of 2 g of concentrate and roughage (wheat straw) in 50:50 ratio. Both the roughage and concentrate were first dried at 100 ± 2 °C for 24 h and finely grounded. Rumen contents (liquor and fiber) were collected from freshly slaughtered goats at a local slaughterhouse. The rumen contents were transferred to pre-warmed (39±1 °C), anaerobic containers to maintain microbial viability. It was then transported to the laboratory and flushed with CO_2_ and kept at appropriate temperature (39 °C). The first trial was conducted on 1 L bioreactor vessel and total operating time was observed.

In the trial using the 1 L vessel, no substrate replacement was carried out, and the dilution rate was maintained at 0.40 ml/min. This vessel could accommodate only 500 ml of rumen liquor (with a maximum capacity of 800 ml). In contrast, the 2.5 L vessel allowed regular substrate replacement, had a lower dilution rate of 0.29 ml/min, and supported 1 L of rumen liquor (with a maximum capacity of 2 L). After conducting trial on 1 L bioreactor vessel, another trial was conducted in 2.5 L bioreactor vessel. Total operating time was observed. Parameters recorded included pH, net gas production, methane production, motility, protozoa count and dry matter degradation.

### Economic considerations

A comprehensive breakdown of the costs associated with the developed apparatus was calculated and analyzed for cost effectiveness.

## Method validation

### Structural modifications and integrity

**Fig. 1:**
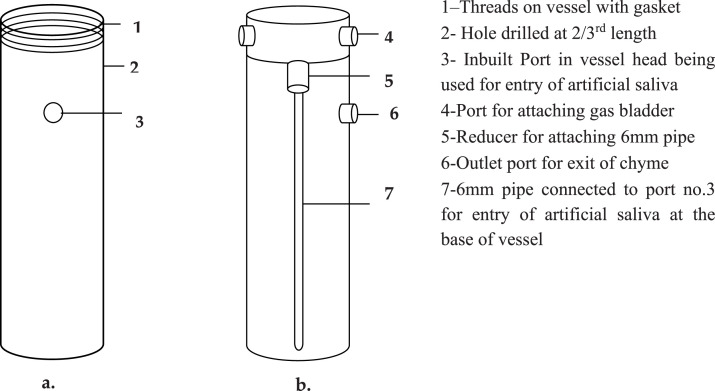
Line Diagram depicting modifications made to the 1 L vessel, a) Front view; b).

Bioreactor vessels, used for fermentation were integral for fermentation, specifically designed for maintaining controlled rumen environments during microbial, enzymatic, or cellular processes. The shape and size of culture containers significantly influence the growth rate of cultures by affecting gaseous diffusion. Factors such as cost, reusability, and washing requirements were also the critical considerations when selecting bioreactor containers to ensure they do not negatively impact in vitro growth. Modifications to the acrylic vessel allowed leak-proof operation, anaerobic integrity, and easy access to internal components for sampling and substrate replacement. The modular design also simplified cleaning and reuse. The modifications made are shown in Fig. 1, Fig. 2, Fig. 3, Fig. 4. To develop an effective in vitro continuous rumen fermentation system, structural modifications were made to both the 1 L and 2.5 L bioreactor vessels to support liquid outflow, substrate addition, mixing, and gas handling. A hole (3) was drilled and threaded at two-thirds the height (7 inches from the bottom) of the 1L vessel, accommodating 860 ml of liquid, to serve as the chyme outlet. A connector (6) was fixed with an O-ring/Teflon tape to ensure a leak-proof seal (Fig. 1). The vessel cap was equipped with connectors (4) for artificial saliva input (via peristaltic pump), gas outlet, and a USB-powered submersible mixer. The process of drilling, threading, and fitting for the 1 L vessel is illustrated with its layout in Fig. 1.

**Fig. 2:**
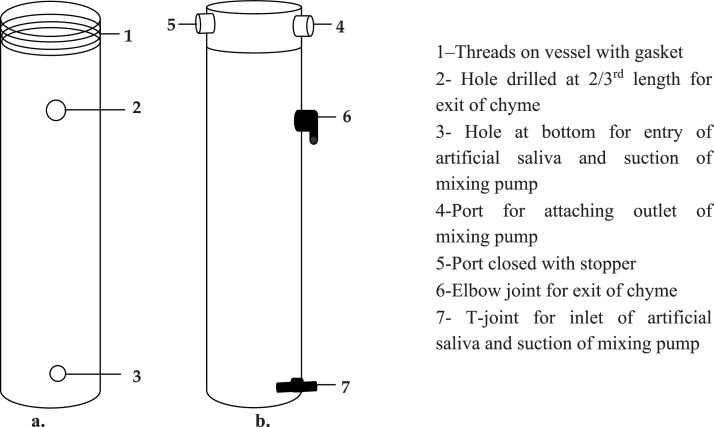
Line Diagram and an image of modified bioreactor vessel (a) Front view, (b) Side view, (c) Image of 2.5 L vessel with connectors.

**Fig. 3:**
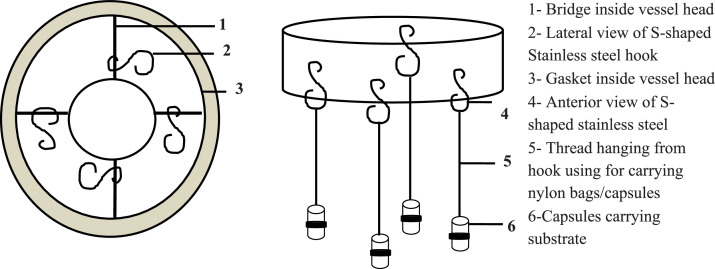
Line diagram of cap of 1 L bioreactor vessel depicting hooks inserted inside the cap bridges, a) Inside view of cap; b) Lateral view of cap with capsules hanging by nylon threads tied to hooks.

**Fig. 4:**
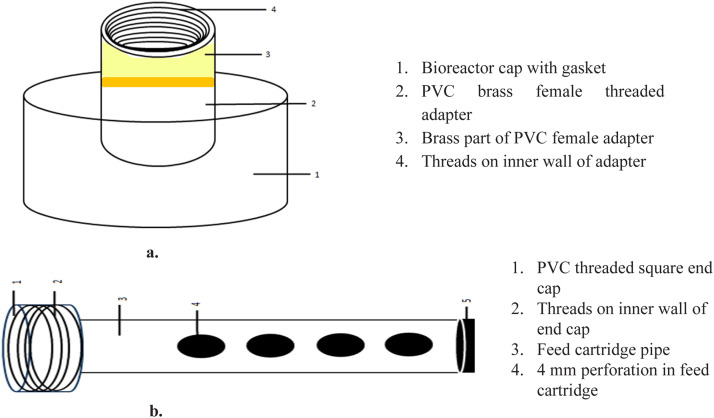
Line diagram of cap and feed cartridge, a) Perspective view bioreactor’s cap modified and fixed with PVC brass female adapter b) Perspective view of feed cartridge unit consisting of perforated PVC pipe fixed with end cap on one end and Teflon stopper on the other.

Similarly, in the 2.5L vessel, a hole was drilled 1 inch above the base (3) to introduce artificial saliva through a T-joint connected to both a peristaltic and mixing pump (Fig. 1). Modifications to the 2.5 L vessel body, including the port fittings, are shown in Fig. 2. To facilitate in-experiment substrate handling, further changes were made to the vessel caps. In the 1L vessel, S-shaped stainless steel hooks (2 & 4) were inserted into small holes pierced in the cap bridges, enabling suspension of nylon-threaded (5) substrate capsules (6). However, this design limited mid-experiment access and posed a risk of disrupting anaerobic conditions (Fig. 3).

These limitations were addressed in the 2.5L vessel cap by installing a 47 mm threaded central hole fitted with a lightweight PVC brass female adapter. A feed cartridge unit comprising a perforated PVC pipe, end cap, and removable Teflon stopper was inserted through this adapter (Fig. 4). This allowed easy insertion/removal of substrate bags without disturbing system integrity. The feed cartridge system and cap modifications are shown in Fig. 4.

### Temperature and flow stability

A temperature regulation unit was successfully developed to simulate the rumen environment by maintaining a stable temperature of 39±1 °C within the bioreactor. The unit was constructed using a thermostat sensor configured and calibrated to cutoff at 39.5 °C temperature with a wait time of 0.5 °C to avoid frequent switching. The heating unit was designed to switch on when the temperature fell below 39 °C and switch off at 39.5 °C, maintaining a consistent range [4]. The configured settings remain constant unless manually adjusted, eliminating the need for regular recalibration unless a temperature change is required. The dry-box heating method provided excellent temperature control within 0.5 °C. Flow rate calibration ensured consistent buffer supply, and cyclic motility pumps prevented sedimentation. This setup was suitable for further experimentation and application in simulating the rumen environment in vitro.

The artificial saliva delivery system was successfully developed and implemented to provide a precise, controlled, and continuous flow of artificial saliva from the storage tank to the bioreactor. This system proved to be a reliable solution for maintaining optimal environmental conditions for microbial or cell culture growth in the bioreactor. The delivery system was constructed using a peristaltic pump, polyurethane tubing, and connectors. Neumann et al. [5], Williams and Wick [4], and Mason [6] successfully utilized peristaltic pumps for the precise addition of buffer or media in fermentation systems. The airtight union ensured no leakage or contamination, maintaining sterility within the bioreactor. The inclusion of a speed controller and timer module allowed precise control of the flow rate and timing of saliva delivery as given in Table 1.

**Table 1:** Artificial saliva flow rate with peristaltic pump connected with speed controller and timer module.

**On time (OP), seconds**	**Off time (CL), seconds**	**Amount of water displaced, ml (a)**	**Total time of operation, hours (b)**	**Flow rate, ml/hour (a/b)**	**Flow rate, ml/minute**
10	100	500	9.50	52.63	0.87
10	150	500	10.0	50.0	0.83
10	180	500	13.75	36.36	0.60
10	220	500	15.00	33.33	0.55
10	250	500	18.93	26.41	0.44
10	300	500	28.50	17.54	0.29
10	360	500	36.0	13.88	0.23

For further trials flow rates of 0.44 ml/min and 0.29 ml/min was selected for 1 L vessel and 2.5 L bioreactor vessel, respectively, based on the dilution rate which were tested on 1 L and 2.5 L vessel, respectively and previous studies [5 & 10].

### Motility simulation system

To replicate the reticulorumen motility, different pumping systems were evaluated. A submersible pump was initially tested and functioned well in water but performed poorly with rumen liquor due to its higher viscosity. A mini vibrating motor was also trialed; however, the vibration generated was insufficient relative to the vessel volume, leading to inadequate mixing. The diaphragm pump was found to be the most suitable, as it worked efficiently without requiring submersion inside the rumen liquor.

All variable pumps and motors tested for rumen motility were connected to a speed controller and timer module, set to simulate the natural rumen motility rate of two contractions per minute [7]. This intermittent approach effectively replicated natural rumen motility and introduced an innovative aspect not present in techniques like IVGPT. Arowolo et al. [8] used similar mechanism where mixing was controlled by a computer-operated step motor and it automatically rotated every minute for 30 s [8]. Muetzel et al. [9] and Mason [6] also reported that intermittent mixing was more effective than continuous mixing of rumen contents, as it better replicated natural rumen conditions. On the other hand, some reported a complete disappearance of protozoa after 7 days of incubation in a continuous fermentation system which was constantly homogenized.

Among the tested methods, the diaphragm pump most accurately mimicked rumen motility, closely maintaining natural conditions without getting blocked. It worked continuously throughout the trial. Rumen motility plays a pivotal role in ensuring the continuous microbial inoculation of ingesta, a critical process for effective rumen fermentation [9]. The addition of a mixing pump operating at a rate mimicking natural rumen motility has been shown to aid in the even distribution of microbes within the rumen. This mechanism promoted efficient breakdown of feed particles, ensured consistent fermentation activity, and maintained an optimal environment for microbial processes, enhancing the overall efficiency of fermentation systems. The results of which was confirmed using preliminary trial (section 3.5) Table 3.

### Gas production

The three gas collection units tested are compared in Table 2 below. Out of all variables selected for gas collection unit, rubber latex bladder was found to be most suitable for gas collection trial. Bhatta et al. [10] and Czerkawski and Breckenridge [11] used gas proof bags for collection of gas during continuous rumen fermentation experiments.

**Table 2:** Comparison of gas collection unit for continuous rumen bioreactor (CRB) trials.

**S.No.**	**Gas collection unit**	**Merits**	**Limitations**	**Selected (Yes/No)**
1.	Rubber Balloon	Inexpensive, high availability	Fragile, leakage was reported, less reliable	No
2.	Polyvinyl pouches (1 L and 5 L)	Tightly sealed body, strong material	Low availability, rigid, less flexibility	No
3.	Latex bladder	Expandable/flexible in nature, long nozzle, no leakage	-	Yes

### Preliminary fermentation trial

Trials were conducted to evaluate the performance of the bioreactor and its components using different parameters, which confirmed that the system was functioning effectively. The results of the preliminary trial for determining the operating period in 1 L and 2.5 L vessel are given in Table 3, Table 4.

**Table 3:** Parameters observed in 1 L bioreactor vessel over the period of 7 days trial.

**Parameters**	**Day 1**	**Day 3**	**Day 5**	**Day 7**	**SEM**	**p-value**
pH	7.1	6.7	7.2	7.5	0.3	1.3
Total gas production, ml/g DM	45	61	52	0	3.45	0.65
Methane production, ml/g DM	4.12	5.14	3.58	0	0.54	0.31
MBRT, minutes	<3 min	<6 min	< 8 min	> 10 min	-	-
Motility	+++	++	+	+	-	-
Protozoa count, *N* × 10^4^/ml	11.5^b^	8.61^b^	4.80^a^	1.25^a^	0.41	0.04
Dry matter degradability, %	-	-	-	40.5	2.02	-

abValues within rows with different superscripts differ significantly (*P* < 0.05).

**Table 4:** **Parameters observed in 2.5L bioreactor vessel over the period of 7 days trial.**

**Parameters**	**Day 1**	**Day 10**	**Day 20**	**Day 30**	**Day 40**	**SEM**	**p-value**
pH	7.0	6.7	7.1	6.9	6.9	0.01	1.2
Total gas production, ml/g DM	150.20^a^	155.73^a^	200.21^b^	210.15^ab^	190.40^ab^	5.21	0.03
Methane production, ml/g DM	32.60	30.45	35.64	31.86	28.74	0.98	0.50
MBRT, minutes	<3min	< 6 min	< 6 min	< 6 min	< 10 min	-	-
Motility	+++	++	++	++	+	-	-
Protozoa count, *N* × 10^4^/ml	13.45^b^	4.73^a^	5.01^a^	4.68^a^	4.32^a^	0.75	0.02
Dry matter degradability, %	40.50	43.5	44.0	41.0	39.50	1.72	0.61

abValues within rows with different superscripts differ significantly (*P* < 0.05).

The system maintained stable microbial activity, pH, and gas production, and methane output confirmed active fermentation and microbial viability. The 1 L vessel could not maintain a stable environment beyond 7 days, whereas the 2.5 L bioreactor sustained stability for up to 40 days owing to its larger rumen liquor volume, slower saliva dilution rate, and regular substrate replacement, which together supported a stable microbial environment.

### Cost-effectiveness and applicability

In the field of animal nutrition, effective rumen studies are crucial for optimizing livestock health and productivity. However, traditional methods often face challenges such as limited operational periods, high costs, and insufficient real-time data monitoring. The Continuous Rumen Bioreactor (CRB) addresses these limitations by providing a continuous, economically viable solution for in vitro rumen studies. Designed to operate efficiently over prolonged periods, the CRB not only reduces operational costs but also minimizes resource consumption compared to existing techniques. The itemized cost of each component used in the system is presented in **Table A1 (Appendix)** for full transparency and reproducibility

Traditional methods, such as In vitro Gas Production Technique (IVGPT) and Rumen Simulation Technique (RUSITEC), can incur substantial costs due to their resource-intensive nature and limited operational efficiency. For instance, IVGPT often requires multiple setups and extensive monitoring, leading to cost in range of Rs. 4,15,500 to Rs. 1246,500, approximately. Whereas, the basic setup of RUSITEC can cost between Rs. 831,000 to Rs. 2493,000, approximately. In contrast, the CRB was designed to operate continuously and economically, potentially reducing operational expenses by approximately 90 %, thus making it a more accessible option for researchers and farmers. A summary comparison between the total cost of the developed CRB and the estimated market price of a similar-capacity commercial system is presented in Table 5. While the developed unit cost was only INR 43,879, commercial alternatives typically cost around INR 3,00,000, leading to an estimated 85.4 % reduction in expenditure. This substantial saving makes the CRB accessible to smaller laboratories, academic institutions, and research stations with limited budgets.

**Table 5:** Cost comparison between developed CRB and commercial continuous fermentation systems.

**Parameter**	**Developed CRB (INR)**	**Commercial System (INR)**	**Savings (INR)**	**% Cost Reduction**
Total system cost	43,879	3,00,000*	2,56,121	85.4 %

*Approximate market price for similar capacity and functionality.

### Continuous rumen bioreactor (CRB) operation

The Continuous Rumen Bioreactor (CRB) system consists of two modified fermentation vessels (16,36) housed in a thermostatically controlled incubator chamber (49). The system is designed to maintain conditions similar to the rumen environment, with continuous supply of artificial saliva, mixing, chyme removal, and gas collection.

Artificial saliva is stored in a dedicated tank (1) fitted with a head assembly (5) that includes ports for CO₂ supply (2), an airlock valve (3), and a USB-powered submersible pump connection (4). Saliva is delivered via polyurethane pipes (7) through T-union joints (8) and three-way cannula valves (9, 13, 31, 32) to the bioreactor vessels. Each bioreactor has a peristaltic pump (11, 33) controlling saliva inflow through inlet silicone pipes (10, 34) and outlet pipes (12, 35), ensuring regulated flow rates.

Inside each vessel, a feed cartridge (17, 37) containing nylon bags or capsules with feed substrates is suspended. The cartridges have perforations (18, 38) to allow exchange with rumen liquor. The top of each vessel is sealed with a cap assembly (19, 39) containing PVC brass adapters (20, 40) and threaded end caps (21, 41) to enable insertion or removal of the feed cartridge without disrupting anaerobic conditions. Motility pumps (22, 33) connected via plastic joints (27–30) circulate fluid, simulating rumen motility.

Digested chyme exits each vessel through side ports (26, 42) fitted with threaded elbow joints (27, 43) and flows into chyme collection vessels (43, 47), each sealed with a gasketed cap (44, 45) and equipped with tap joints (46) for sampling.

Gas produced during fermentation is collected via ports at the vessel caps and stored in latex gas bladders (42, 48) for measurement.

The incubator’s control panel (50) houses the thermostat display (51), timer modules for motility and saliva pumps (52–55, 57–60), and speed controllers (56, 58, 59, 61) to fine-tune pump speeds. Switches (62–65) control bulbs, LED indicators, and main system power.

This integrated design as shown in Fig. 2A allows stable temperature control, continuous substrate feeding, and uninterrupted removal of fermentation end products, closely mimicking in vivo rumen fermentation while enabling controlled laboratory experimentation.

Detailed description of Fig. 2 A

1. Artificial saliva tank
2. Silicone pipe and glass tube for CO2 cylinder
3. Airlock Valve
4. USB port for submersible pump
5. Cap/head of artificial saliva tank
6. Stopcock
7. Polyurethane pipe
8. T union joint
9. Three way cannula valve with reducer for bioreactor 1
10. Inlet Silicon pipe of peristaltic pump
11. Peristaltic pump 1 (for bioreactor 1)
12. Outlet Silicon pipe of peristaltic pump
13. Three way cannula valve with reducer
14. T-type male threaded connector
15. Bioreactor 1vessel
16. Feed cartridge of bioreactor 1
17. Perforations in feed cartridge
18. Lock of feed catridge
19. Bioreactor cap with gasket
20. PVC brass female threaded adapter
21. PVC threaded square end cap
22. Motility pump 1 (for bioreactor 1)
23. Contents of bioreactor
24. Male Threaded Elbow joint
25. Silicon pipe
26. Hole in bioreactor for chyme exit
27. Plastic male Threaded Elbow joint
28. Silicon pipe
29. Plastic elbow joint
30. Silicon pipe
31. 3 way cannula valve with reducer for bioreactor 2
32. 3 way cannula valve with reducer
33. Peristaltic pump 2 (for bioreactor 2)
34. Plastic T-type male threaded connector
35. Bioreactor 2 vessel
36. Feed cartridge 2 (of bioreactor 2)
37. Male Threaded Elbow joint
38. Plastic male Threaded Elbow joint
39. Motility pump 2 (for bioreactor 2)
40. Plastic elbow joint
41. Silicon pipe
42. Plastic Male threaded elbow joint
43. Chyme/ Digesta vessel 1(for bioreactor 1)
44. Cap of chime vessel with gasket
45. Plastic male threaded tap joint
46. Gas collection bladder 1 (for bioreactor 1)
47. Chyme vessel 2 (for bioreactor 2)
48. Gas collection bladder 2 (for bioreactor 2)
49. Incubator chamber
50. Control panel
51. Thermostat or temperature display
52. Timer module for motility (for bioreactor 1)
53. Timer module for saliva (for bioreactor
54. Speed controller for motility (for bioreactor 1)
55. Speed controller for saliva (for bioreactor 1)
56. Timer module for motility (for bioreactor 2)
57. Timer module for saliva (for bioreactor 2)
58. Speed controller for motility (for bioreactor 2)
59. Speed controller for saliva (for bioreactor 2)
60. Switch of bulb 1
61. Switch of bulb 2
62. LED switch
63. Main power switch
64. Power switch for bioreactor 1
65. Power switch for bioreactor 2

**Fig. 2 A:**
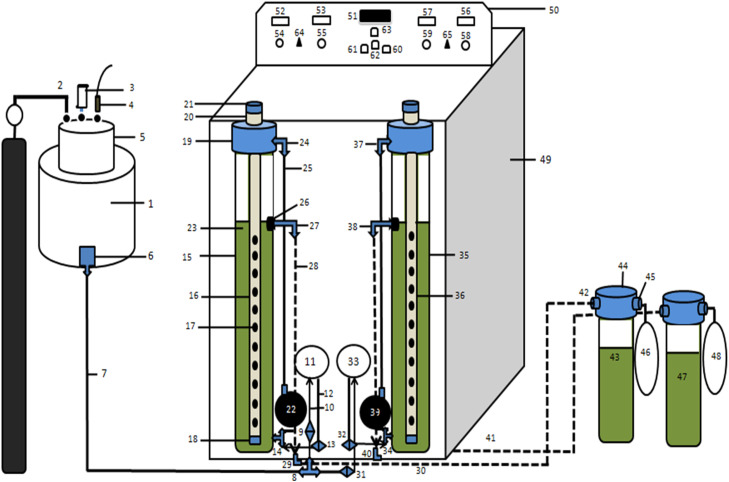
**.** Schematic view (line diagram) of Continuous Rumen Bioreactor (CRB) for rumen studies.

#### Comparison of rusitec and the developed CRB

The Rumen Simulation Technique (RUSITEC) is a well-established in vitro continuous fermentation system widely used for studying rumen function. While RUSITEC offers robust control over experimental variables, it has limitations in terms of cost, accessibility, and certain operational aspects. The developed CRB addresses several of these limitations while maintaining comparable functionality (Fig. 2B).

**Table:** 

**Feature**	**RUSITEC**	**Developed CRB**
**Cost**	High (∼INR 10–15 lakh)	Low (INR 43,879)
**Scale**	Generally larger volume (800–1000 ml vessels)	Flexible vessel sizes (1 L and 2.5 L)
**Feed handling**	Nylon bags suspended inside vessel; replacement requires vessel opening	Feed cartridge system allows substrate addition/removal without disrupting anaerobic conditions
**Temperature control**	Water bath or heated chamber	Thermostatically controlled incubator with 100 W bulbs and fans
**Saliva delivery**	Peristaltic pump	Peristaltic pump
**Chyme removal**	Manual or automated	Continuous outlet at 2/3 vessel height to chyme collection container
**Customizability**	Limited due to fixed commercial design	High; components fabricated locally and easily modified
**Operational complexity**	Requires technical expertise and fixed infrastructure	Simple to operate, repair, and scale for different experimental designs

**Fig. 2B:**
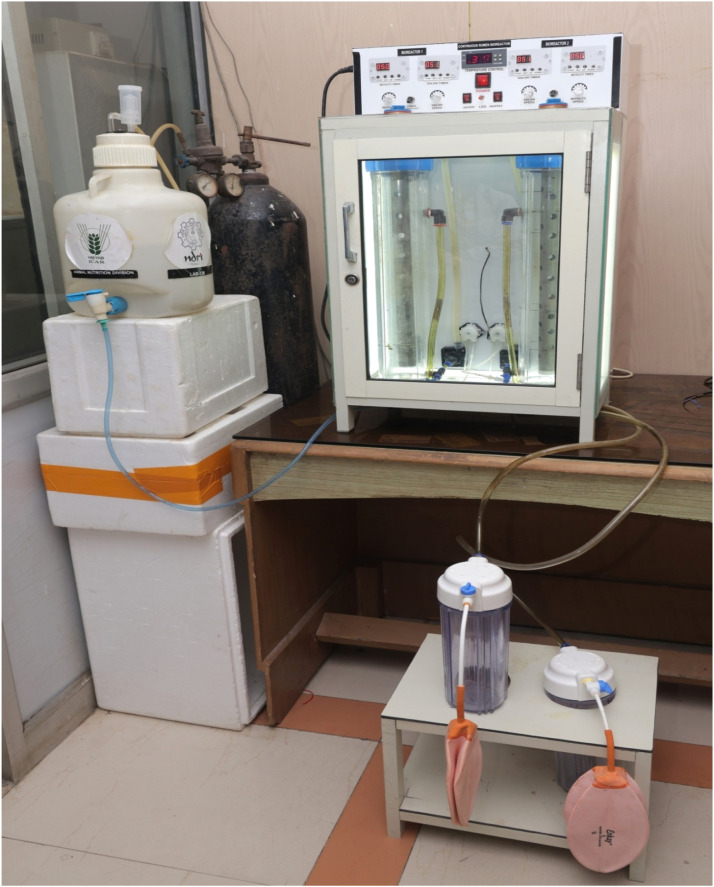
Schematic view of Continuous rumen bioreactor (CRB) arranged with all its components.

While our Continuous Rumen Bioreactor (CRB) study focuses on ruminal fermentation and nutrient utilization, the outcomes of Harahap et al. highlight the relevance of exploring bioactive feed additives—such as algae—in modulating fermentation end-products [11]. This reinforces the value of adaptable, in vitro systems like CRB for assessing novel dietary strategies under controlled conditions.

The ability of the CRB to maintain stable fermentation conditions makes it suitable for evaluating non-conventional feeds, such as cassava, whose in vivo benefits and impacts on rumen health have been documented in meta-analyses [1]. This approach can reduce the cost and time associated with large-scale animal trials while allowing precise control over fermentation parameters

## Conclusions

The continuous rumen bioreactor (CRB) was successfully developed and standardized using a 2.5 L vessel, with stable operation achieved at a saliva flow rate of 0.29 ml/min and a motility rate of 2 cycles/minute. Key components, including a diaphragm pump for mimicking rumen motility, a latex bladder for gas collection, and a thermostat-regulated incubator with separate compartments for bioreactor, heating, and control, ensured reliable performance without leakage. The system maintained functionality for 40 days with regular substrate replacement and recharging with fresh fiber, demonstrating its suitability for simulating rumen fermentation under controlled conditions.

**Limitations:** Although the CRB was validated for up to 40 days, longer operation and scalability to larger vessels were not tested and remain to be investigated.

**Ethics approval and consent to participate:** NA

## Credit authors statement

**Harneet Kour:** Conceptualization, Methodology, Investigation (development and evaluation of CRB), Data curation, Formal analysis, Writing- original draft, review & editing. **Raman Malik:** Supervision, Methodology, Validation, Writing – review & editing. **Chander Datt:** Validation, Data curation, Writing – review & editing. **Parul Rana:** Data curation, Formal analysis, Writing – review & editing.

**Availability of data and material:** All data generated or analyzed during this study are included in this published article. The data supporting the findings of this study are available from the corresponding author upon reasonable request.

## Declaration of competing interests

The authors declare that they have no known competing financial interests or personal relationships that could have appeared to influence the work reported in this paper.

## Figures and Tables

**Table A1:** Detailed cost of components used in the developed CRB.

**S. No.**	**Item/Component**	**Number of Units**	**Cost per Unit (INR)**	**Total Cost (INR)**
1	Speed Controller	4	499	1996
2	Timer Modules	4	150	600
3	Thermostat	1	2250	2250
4	DC fans	4	80	320
5	Bulbs (100 W)	2	20	40
6	Polyurethane pipe (8 mm)	1 m	30	30
7	Polyurethane pipe (6 mm)	1 m	20	20
8	Polyurethane pipe (9.5 mm)	5 m	40	200
9	Connectors/Joints	10	40	400
10	Filter vessel (bioreactor) 2.5L	2	3500	7000
11	Filter vessel (chyme) 1L	2	1500	3000
12	Three-way cannula valve	4	40	160
13	Latex gas bladder	2	450	900
14	Incubator box	1	20,000	20,000
15	Peristaltic pump (saliva)	2	678	1356
16	Diaphragm pumps (motility)	2	500	1000
17	Centrifugal pump (mixing of saliva)	1	500	500
18	PVC pipe 1.25″ (Feed cartridge)	1 m	60	60
19	PVC female brass threaded adapter	2	515	1030
20	PVC threaded square end cap	2	60	120
21	Airlock valve	1	279	279
22	Bulb holder	2	20	40
23	12 V Adapter (for artificial saliva mixing pump USB)	1	100	100
24	USB wire	1	10	10
25	Switches	6	30	180
26	Wires	10 m	20	200
27	Wire connectors	10	108	1088
28	Miscellaneous	–	–	1000
**Total**				**43,879**

## Data Availability

Data will be made available on request.

## Appendix

Table A1.
